# Characteristics Associated With Multisystem Inflammatory Syndrome Among Adults With SARS-CoV-2 Infection

**DOI:** 10.1001/jamanetworkopen.2021.10323

**Published:** 2021-05-19

**Authors:** Giovanni E. Davogustto, Daniel E. Clark, Edward Hardison, Ahmad H. Yanis, Brandon D. Lowery, Natasha B. Halasa, Quinn S. Wells

**Affiliations:** 1 Division of Cardiovascular Medicine, Department of Medicine, Vanderbilt University Medical Center, Nashville, Tennessee; 2 Department of Medicine, Vanderbilt University Medical Center, Nashville, Tennessee; 3 Department of Pediatrics, Vanderbilt University Medical Center, Nashville, Tennessee; 4 Vanderbilt Institute for Clinical and Translational Research, Vanderbilt University Medical Center, Nashville, Tennessee; 5 Department of Pharmacology, Vanderbilt University Medical Center, Nashville, Tennessee; 6 Department of Biomedical Informatics, Vanderbilt University Medical Center, Nashville, Tennessee

## Abstract

This cohort study describes the clinical characteristics and outcomes of patients with multisystem inflammatory syndrome among adults with laboratory-confirmed SARS-CoV-2 infection at a single US tertiary care medical center.

## Introduction

A postacute COVID-19 multisystem inflammatory syndrome (MIS) has been recognized as a rare, yet severe, complication of SARS-CoV-2 infection. First characterized in children,^1,2^ MIS in adults (MIS-A) has now been reported,^3^ leading to the publication of a working case definition by the Centers for Disease Control and Prevention.^4^

The goal of this cohort study was to describe the spectrum of MIS-A presentation after SARS-CoV-2 infection. We identified cases of MIS-A among all adults with laboratory-proven subacute or convalescent SARS-CoV-2 infection at a single tertiary care medical center and described their clinical characteristics and outcomes.

## Methods

This single-center, retrospective cohort study was conducted at Vanderbilt University Medical Center, Nashville, Tennessee. Adults at risk of MIS-A were identified from those hospitalized with positive SARS-CoV-2 test results. The overview of cohort identification and patient selection is described in eFigure 1 in the Supplement. Briefly, we identified all adults 21 years or older with a positive SARS-CoV-2 test result and a subsequent admission from March 1 through September 30, 2020. Those with laboratory-confirmed SARS-CoV-2 infection who were admitted either (1) between 14 and 84 days after positive SARS-CoV-2 reverse transcriptase–polymerase chain reaction test results (RT-PCR) or (2) 15 days before or after positive SARS-CoV-2 serologic test results were classified as being at risk for MIS-A (eFigure 2 in the Supplement). Patients admitted more than 84 days after positive SARS-CoV-2 RT-PCR test results were excluded. All others were classified as acute COVID-19 admissions for comparison. The study protocol was approved by the Vanderbilt University Medical Center Institutional Review Board. Informed consent was not required as the study met exemption based on 45 CFR §46.104(d) category (4)(iii). This study followed the Strengthening the Reporting of Observational Studies in Epidemiology (STROBE) reporting guideline.

Demographic characteristics (patients’ age, sex, race, and ethnicity) and preexisting chronic comorbid conditions (eMethods and eTable 1 in the Supplement) captured during clinical encounters were extracted from Vanderbilt University Medical Center Research Derivative^5^ and compared between groups. The electronic medical records of individuals at risk for MIS-A were manually reviewed and presented by 1 of 3 authors (G.E.D., D.E.C., or E.H.) and then adjudicated by consensus among the three. Organ system involvement was assessed using previously established criteria (eTable 2 in the Supplement).^1^ Continuous variables were compared using the Mann-Whitney test, and categorical variables were compared with the Fisher exact test. Tests were 2-tailed, and *P* < .05 was considered significant. Data analyses were performed using R software version 4.0.2 (R Foundation for Statistical Computing).

## Results

A total of 7196 patients with evidence of SARS-CoV-2 infection by RT-PCR or serologic testing were identified from Vanderbilt University Medical Center electronic health records. Of those, 839 patients (11.7%) were admitted with a positive SARS-CoV-2 test result during the study’s time frame. Of those admitted, 156 patients (11.7%) were classified as being at risk for MIS-A, and 683 (81.4%) were classified as having acute COVID-19. After adjudication, 15 of the 156 patients (9.6%) at risk met criteria for MIS-A^4^; the remaining 141 patients (90.3%) were excluded from the analyses.

Among the 698 patients included in this analysis, the median age was 55.8 years (range, 21.2-96.9 years), 372 (53.3%) were men, 326 (46.7%) were women, 406 (58.2%) were White individuals, and 169 (24.2%) were Black individuals. Additional patient characteristics are provided in the Table. Patients with MIS-A were younger (median age, 45.1 years; range 21.3-84.0 years vs 56.5 years; range, 21.2-96.9 years for patients admitted for acute COVID-19 symptoms; *P* = .02) and more likely to have evidence of SARS-CoV-2 infection documented by serologic testing (9 patients [60.0%] with MIS-A vs none with COVID-19; *P* < .001). Other demographic characteristics and comorbidities did not differ from those of patients requiring admission for acute SARS-CoV-2 infection.

**Table.** Demographic Characteristics of Patients According to Type of SARS-CoV-2–Related Admission

Characteristic	Type of admission, No. (%)		*P* value
	Acute COVID-19 (n = 683)	MIS-A (n = 15)	
Age, median (range), y	56.5 (21.2-96.9)	45.1 (21.3-84.0)	.02
Sex			
Female	321 (47.0)	5 (33.3)	.43
Male	362 (53.0)	10 (66.7)	
Self-reported race			
Asian	18 (2.6)	2 (13.3)	.07
Black	165 (24.2)	4 (26.7)	
White	403 (59.0)	3 (20.0)	
Other or unknown^a^	97 (14.2)	6 (40.0)	
Self-reported ethnicity			.95
Hispanic	88 (12.9)	2 (13.3)	
Non-Hispanic	561 (82.1)	12 (80.0)	
Unknown	34 (5.0)	1 (6.7)	
Underlying comorbid conditions^b^			
Any	522 (76.4)	10 (66.7)	.57
Hypertension	173 (25.3)	3 (20.0)	.86
Coronary artery disease	94 (13.8)	1 (6.7)	.68
Heart failure	55 (8.1)	0	.51
Diabetes mellitus	249 (36.5)	3 (20.0)	.30
Cancer	169 (24.7)	4 (26.7)	>.99
Inflammatory	19 (2.8)	0	>.99
COPD	55 (8.1)	0	.51
Chronic kidney disease	101 (14.8)	3 (20.0)	.85
Obesity (BMI >30)	289 (42.3)	5 (33.3)	.66
No. of comorbid conditions, median (range)	1 (0-8)	1 (0-5)	.22
BMI, median (range)	29.01 (14.4-72.8)	29.2 (20.1-39.2)	.80
Length of admission, median (range),d^c^	5 (0-73)	4 (2-10)	.44
Diagnostic method			
RT-PCR	683 (100)	6 (40.0)	<.001
Serologic testing	0	9 (60.0)	

Abbreviations: BMI, body mass index (calculated as weight in kilograms divided by height in meters squared); COPD, chronic obstructive pulmonary disease; MIS-A, multisystem inflammatory syndrome in adults; RT-PCR, reverse transcriptase polymerase chain reaction.

^a^ Other races were Pacific Islander and Native American.

^b^ The listed underlying comorbidities were extracted from the electronic health records using diagnostic and procedure codes specified in eTable 1 in the Supplement.

^c^ For patients with MIS-A who also had an acute COVID-19 admission, length of admission represents that of MIS-A admission only.

The clinical characteristics of MIS-A cases are presented in the Figure, A. Nine of the 15 patients with MIS-A (60.0%) had acute COVID-19 symptoms, and 3 (20.0%) required admission for acute COVID-19 before being admitted for MIS-A. For patients with prior admission for acute COVID-19, the median interval between acute COVID-19 admission and MIS-A admission was 23 days (interquartile range [IQR], 16.0-24.5 days). During MIS-A admission, 5 patients (33.3%) required intensive care treatment for hemodynamic monitoring (n = 3), vasopressor support (n = 1), or noninvasive ventilatory support (n = 1). Furthermore, during MIS-A admission, 3 patients (20.0%) had MIS-A as a clinical diagnosis, 4 (26.7%) received immunosuppressive therapy, 7 (46.6%) received antibiotic therapy, and no participants died. Organ system involvement per patient is shown in the Figure, B. The median number of organ systems involved was 4 (IQR, 2.0-4.5). The gastrointestinal, hematologic, and kidney systems were most commonly affected.

**Figure.**
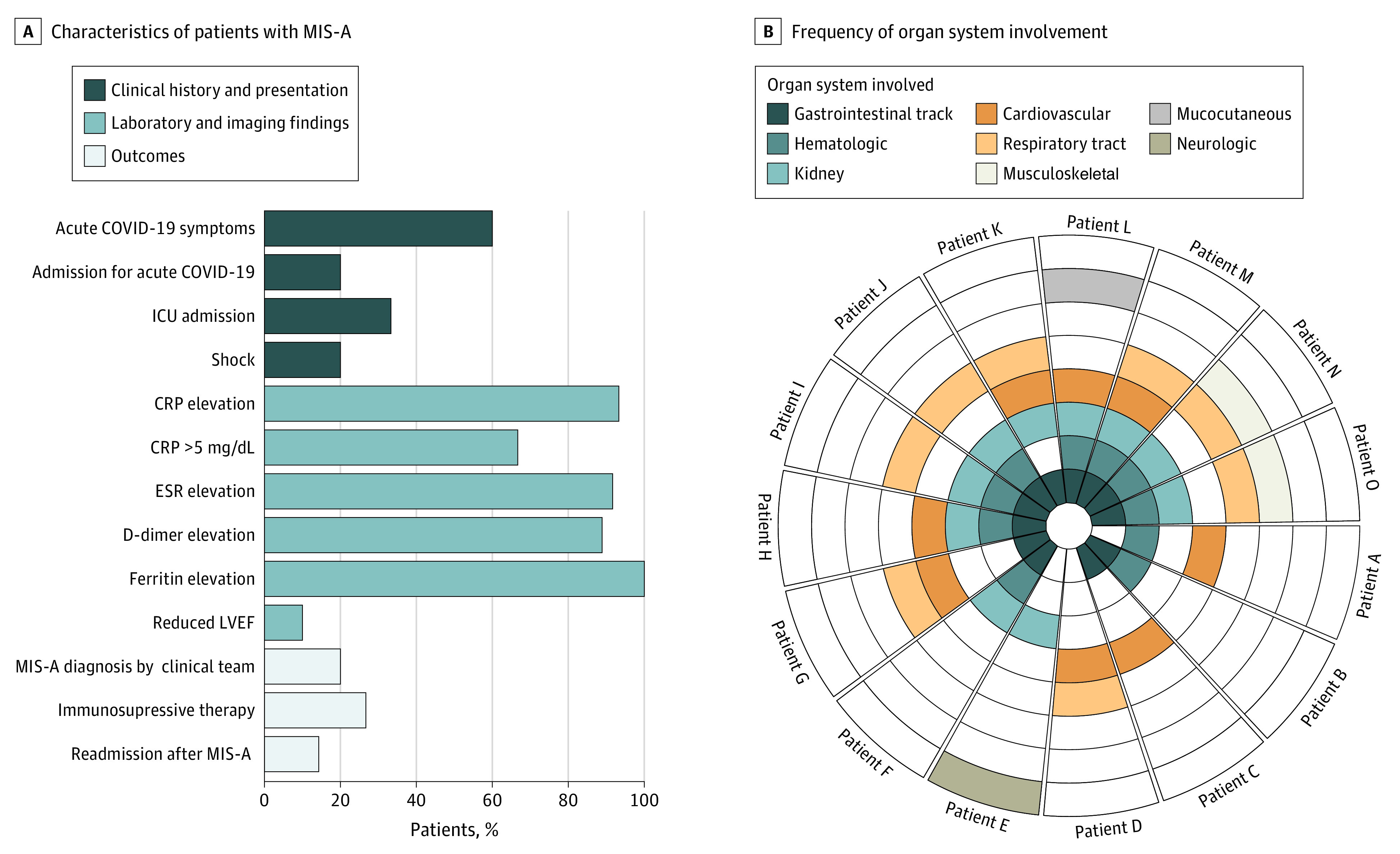
Clinical Presentation and Organ Involvement of Patients Identified With Multisystem Inflammatory Syndrome in Adults (MIS-A) A, Data were available for left ventricular ejection fraction (LVEF) from 10 patients, for erythrocyte sedimentation rate (ESR) from 12, for D-dimer level from 9; and for ferritin level from 8. To convert C-reactive protein (CRP) level from milligrams per deciliter to milligrams per liter, multiply by 10. B, Each wedge represents a single patient, and each concentric ring represents an organ system, organized from inside out by frequency of involvement. ICU indicates intensive care unit.

## Discussion

The patients with MIS-A identified in our cohort have a broader distribution of organ involvement and lower illness severity compared with those in previously published series.^4^ Most patients who met the MIS-A criteria were not identified as such by the primary clinical team. This study had some limitations. Our data likely underestimate the incidence of MIS-A because many patients with COVID-19–related admissions did not have routine comprehensive clinical and laboratory assessments to screen for this syndrome. These data suggest that, although uncommon, MIS-A has a more heterogeneous clinical presentation than previously appreciated and is commonly underdiagnosed. Future investigations, including prospective enrollments, are necessary to improve the diagnostic and treatment approaches for patients with MIS-A.

## Notes

Supplement. **eMethods.** Study Design, Participants, and Case Definitions eReferences. **eTable 1.** Definitions of Chronic Morbidities Extracted From the Electronic Health Record **eTable 2.** Organ System Involvement Definitions **eFigure 1.** Overview of Identification of Patients Meeting Multisystem Inflammatory Syndrome in Adults (MIS-A) Case Definition **eFigure 2.** Identification of Participants at Risk of Multisystem Inflammatory Syndrome in Adults (MIS-A) From the Electronic Health Record
