# Supplementary material for: Characteristics Associated With Multisystem Inflammatory Syndrome Among Adults With SARS-CoV-2 Infection
Source: JAMA Netw Open. 2021 May 19;4(5):e2110323. doi: 10.1001/jamanetworkopen.2021.10323 (PMC8134998; doi:10.1001/jamanetworkopen.2021.10323)
Supplement: Supplement. — eMethods. Study Design, Participants, and Case Definitions eReferences. eTable 1. Definitions of Chronic Morbidities Extracted From the Electronic Health Record eTable 2. Organ System Involvement Definitions eFigure 1. Overview of Identification of Patients Meeting Multisystem Inflammatory Syndrome in Adults (MIS-A) Case Definition eFigure 2. Identification of Participants at Risk of Multisystem Inflammatory Syndrome in Adults (MIS-A) From the Electronic Health Record [file jamanetwopen-e2110323-s001.pdf]

SUPPLEMENTARY ONLINE CONTENT

Davogustto GE, Clark DE, Hardison E, et al. Characteristics associated with multisystem inflammatory syndrome among adults with SARS-CoV-2 infection. *JAMA Netw Open.* 2021;4(5):e2110323. doi:10.1001/jamanetworkopen.2021.10323

eMethods. Study Design, Particiants, and Case Definitions

eReferences.

eTable 1. Definitions of Chronic Morbidities Extracted From the Electronic Health Record

eTable 2. Organ System Involvement Definitions

eFigure 1. Overview of Identification of Patients Meeting Multisystem Inflammatory Syndrome in Adults (MIS-A) Case Definition

eFigure 2. Identification of Participants at Risk of Multisystem Inflammatory Syndrome in Adults (MIS-A) From the Electronic Health Record

This supplementary material has been provided by the authors to give readers additional information about their work.

eMethods. Study Design, Particiants, and Case Definitions

The study protocol was approved by the Vanderbilt University Medical Center (VUMC) Institutional Review Board (IRB#200571).

Study design and participants:

The study was a single-center retrospective cohort design conducted at Vanderbilt University Medical Center (VUMC). Adults ≥ 21 years of age who had evidence of SARS-CoV-2 infection by RT-PCR or serology between March 1st, 2020 and September 30th, 2020 were identified from VUMC Research Derivative. The Research Derivative is a database of clinical and related data derived from the VUMC clinical systems and restructured for research. Data is repurposed from VUMC's enterprise data warehouse, which includes data from StarPanel, VPIMS, and ORMIS (Operating Room Management Information System), EPIC, Medipac, and HEO among others. The medical record number and other person identifiers are preserved within the database. Data types include reimbursement codes, clinical notes and documentation, nursing records, medication data, laboratory data, encounter and visit data, among others. Output may include structured datapoints, such as ICD 9 or 10 codes and encounter dates, semi-structured data such as laboratory tests and results, or unstructured data such as physician progress reports. The database is maintained by the Office of Research Informatics under the direction of Paul Harris, Ph.D.[1]

All patients with testing supporting of SARS-CoV-2 infection were identified from Vanderbilt's Research Derivative (RD). Age, sex, race and ethnicity captured during clinical encounters (either self-reported by patients or administratively assigned into the electronic health record), initial positive SARS-CoV-2 test type and date, date of admission and discharge, BMI, and the presence of chronic comorbidities were extracted from the Electronic Health Record (EHR) using the RD. Race and ethnicity were included due to prior evidence of disproportionate burden of MIS in Black and Hispanic children.[2] Chronic comorbidities extracted included hypertension, coronary atherosclerosis, heart failure, diabetes mellitus, cancer, inflammatory diseases, chronic obstructive pulmonary disease, and chronic kidney disease. These were ascertained from the EHR using ICD9/ICD10/CPT codes, medications, and laboratory values. Definitions used to identify comorbidities are provided in **eTable 1**.

The overview of patient selection is depicted in **eFigure 1**. Adults ≥ 21 years-old at risk of multisystem inflammatory syndrome associated with SARS-CoV-2 infection (MIS-A) were identified as those hospitalized surrounding SARS-CoV-2 testing based on diagnostic method as follows: (**eFigure 2**)

1) RT-PCR group:
    a. Positive SARS-CoV-2 RT-PCR nasopharyngeal swab followed.
    b. Hospital admission ≥ 14 days and ≤ 84 days (12 weeks) from initial positive PCR test.
2) Serology group:
    a. Positive SARS-CoV-2 IgG.
    b. Hospital admission within 15 days before or after positive IgG test.

In the RT-PCR group, a 14-day "blanking period" was implemented to avoid contamination from admissions driven by acute COVID-19 rather than MIS-A. We selected 14 days, because it has been previously suggested that a substantial proportion of MIS-C patients were infected with SARS-CoV-2 at least 1-2 weeks before MIS-C onset.[3] Patients admitted >84 days following positive SARS-CoV-2 RT-PCR were excluded. All others admitted were included in the Acute COVID-19 admission group used for comparison. Although the CDC working definition of MIS-A, defines evidence of prior SARS-CoV-2 infection as any positive test within 12 weeks of symptoms, and does not stipulate a 14-day blanking period from SARS-CoV-2 RT-PCR, we modified this criterium due to the limitations of our retrospective study and potential overlap with acute COVID-19.

© 2021 Davogustto GE et al. *JAMA Network Open.*

For serology, a 15-day window was selected as what the authors thought represented reasonable period for a serology result to be obtained in the onset of MIS-A or to return following potential discharge following MIS-A admission and not be attributable to another systemic illness.

Then, charts of individuals at risk of MIS-A were manually reviewed by one of three physicians in the team (D.E.C., G.E.D., E.H.) to identify charts to discuss in an adjudication group. Patients were excluded during screening if recorded admissions were:

1) Procedural: Delivery, c-section, and other elective procedures.
2) Well-supported alternative diagnosis: For example, sepsis with positive bacterial cultures.
3) Not meeting at least 4 of the 5 MIS-A criteria from the CDC definition.[4]
4) Lack of recovery and progression from COVID-19 initial presentation.

All remaining cases at risk for MIS-A were then presented in a three-physician decision group (comprised by D.E.C., G.E.D., E.H.) and adjudicated as case or excluded. Disagreements were resolved by consensus.

Case classification and definition of organ system involvement:

Cases reviewed in full were adjudicated as MIS-A if the review team agreed that the cases met all 5 MIS-A criteria from CDC's case definition and no alternative explanation of the presentation was plausible.

Organ-system involvement was adjudicated on the basis of symptoms, clinical findings, and laboratory measurements, similarly to previously described[3] and are presented in **eTable 2**.

Data Collection of cases:

Demographics, BMI, and chronic comorbidities for all participants were extracted from the EHR. Definitions used to identify comorbidities are provided in **eTable 1**.

Additional data collected of MIS-A cases included clinical presentation, vital signs on admission, laboratory parameters and microbiologic data obtained during admission, organ system involvement as described above, findings on cardiovascular diagnostic studies, therapies, and survival. Data were obtained from medical records and collected in a Vanderbilt's Research Electronic Data Capture (REDCap) by a member of the research team (D.E.C., G.E.D, E.H., A.Y.).

Statistics:

Data are presented as median and interquartile range for continuous variables, and counts and percentages for categorical variables unless otherwise specified. Missing data were not imputed. Continuous variables were compared using Mann-Whitney U test, and categorical variables compared with Fisher's exact test. Tests were two-tailed. A p-value <0.05 was considered significant. Data analyses were performed and graphs created using R software, version 4.0.2 (R foundation for Statistical Computing, Vienna, Austria).

© 2021 Davogustto GE et al. *JAMA Network Open.*

eReferences.

1. Danciu I, Cowan JD, Basford M, et al. Secondary use of clinical data: the Vanderbilt approach. *J Biomed Inform.* 2014;52:28-35.
2. Lee EH, Kepler KL, Geevarughese A, et al. Race/Ethnicity Among Children With COVID-19-Associated Multisystem Inflammatory Syndrome. *JAMA Netw Open.* 2020;3(11):e2030280.
3. Feldstein LR, Rose EB, Horwitz SM, et al. Multisystem Inflammatory Syndrome in U.S. Children and Adolescents. *N Engl J Med.* 2020;383(4):334-346.
4. Morris SB, Schwartz NG, Patel P, et al. Case Series of Multisystem Inflammatory Syndrome in Adults Associated with SARS-CoV-2 Infection — United Kingdom and United States, March–August 2020. *MMWR Morb Mortal Wkly Rep.* 2020;69:1450–1456.
5. Caforio AL, Pankuweit S, Arbustini E, et al. Current state of knowledge on aetiology, diagnosis, management, and therapy of myocarditis: a position statement of the European Society of Cardiology Working Group on Myocardial and Pericardial Diseases. *Eur Heart J.* 2013;34(33):2636-2648, 2648a-2648d.
6. Section 2: AKI Definition. *Kidney Int Suppl (2011).* 2012;2(1):19-36.
7. Schulman S, Kearon C. Definition of major bleeding in clinical investigations of antihemostatic medicinal products in non-surgical patients. *J Thromb Haemost.* 2005;3(4):692-694.

© 2021 Davogustto GE et al. *JAMA Network Open.*

eTable 1. Definitions of Chronic Comorbidities Extracted From the Electronic Health Record

Comorbidity	Definition
Hypertension	Presence of ANY of the following codes throughout the record:<br>ICD9: 401", "401.*", "402", "402.*", "403", "403.*", "404", "404.*", "405", "405.*"<br>ICD10: "I10", "I10.*", "I11", "I11.*, I12, I12.*, I13, I13.*, I16, I16.*
CAD	Presence of ANY of the following codes throughout the record:<br>ICD9: 410, 410.*, 411, 411.*, 412, 412.*, 413, 413.*, 414, 414.*, V45.82<br>ICD10: I25.1%<br>CPT: 33534, 33535, 33536, 33510, 33511, 33512, 33513, 33514, 33515, 33516, 33517, 33518, 33519, 33520, 33521, 33522, 33523, 92980, 92981, 92982, 92984, 92995, 92996
Heart Failure	Presence of at least one code AND one medication from the list below, AND one BNP (with value > 100 pg/ml)<br>ICD9: 425, 425.*, 428, 428.*<br>ICD10: I42, I42.*, I50, I50.*<br>Medications: "furosemide", "lasix", "bumetanide", "bumex", "torsemide", "demadex", "ethacrynic acid", "edecrin", "metolazone", "zaroxolyn"
Diabetes Mellitus	Presence of ANY of the following codes throughout the record:<br>ICD9: 250, 250.*, V58.67<br>ICD10: Z79.4, Z79.84, E08, E08.*, E09, E09.*, E10, E10.*, E11, E11.*, E13, E13.*
Cancer	Presence of ANY of the following codes throughout the record:<br>ICD9: 140.*-209.*<br>ICD10: C00.*, C01.*, C02.*, C03.*, C04.*, C05.*, C06.*, C07.*, C08.*, C09.*, C10.*, C11.*, C12.*, C13.*, C14.*, C15.*, C16.*, C17.*, C18.*, C19.*, C20.*, C21.*, C22.*, C23.*, C24.*, C25.*, C26.*, C27.*, C28.*, C29.*, C30.*, C31.*, C32.*, C33.*, C34.*, C35.*, C36.*, C37.*, C38.*, C39.*, C40.*, C41.*, C42.*, C43.*, C44.*, C45.*, C46.*, C47.*, C48.*, C49.*, C50.*, C51.*, C52.*, C53.*, C54.*, C55.*, C56.*, C57.*, C58.*, C59.*, C60.*, C61.*, C62.*, C63.*, C64.*, C65.*, C66.*, C67, C68, C69.*, C70.*, C71.*, C72.*, C73.*, C74.*, C75.*, C76.*, C77.*, C78.*, C79.*, C80.*, C81.*, C82.*, C83.*, C84.*, C85.*, C86.*, C87.*, C88.*, C89.*, C90.*, C91.*, C92.*, C93.*, C94.*, C95.*, C96.*, D00.*, D01.*, D02.*, D03.*, D04.*, D05.*, D06.*, D07.*, D08.*, D09.*, D10.*, D11.*, D12.*, D13.*, D14.*, D15.*, D16.*, D17.*, D18.*, D19.*, D20.*, D21.*, D22.*, D23.*, D24.*, D25.*, D26.*, D27.*, D28.*, D29.*.*, D30.*, D31.*, D32.*, D33.*, D34.*, D35.*, D36.*, D37.*, D38.*, D39.*, D40.*, D41.*, D42.*, D43.*, D44.*, D45.*, D46.*, D47.*, D48.*, D49.*
Inflammatory	Presence of ANY of the following codes throughout the record:<br>ICD9: 714, 714.*, 720, 720.*, 725, 725.*<br>ICD10: M0, M1, M2, M3, M4, M5, M6, M7, M8, M9, M10, M11, M12, M13, M14, M15, M16, M17, M18, M19, M20, M21, M22, M23, M24, M25, M26, M27, M28, M29, M30, M31, M32, M33, M34, M35, M36, M37, M38, M39, M40, M41, M42, M43, M44, M45, M46, M47, M48, M49, M50, M51, M52, M53, M54, M55, M56, M57, M58, M59, M60, M61, M62, M63, M64, M65, M66, M67, M68, M69, M70, M71, M72, M73, M74, M75, M76, M77, M78, M79, M80, M81, M82, M83, M84, M85, M86, M87, M88, M89, M90, M91, M92, M93, M94, M95, M96, M97, M98, M99
COPD	Presence of two or more of listed codes AND ANY of the medications from the list below throughout the record:<br>ICD9: 491, 491.0, 491.1, 491.2, 491.20, 491.21, 491.22, 491.8, 491.9, 492, 492.0, 492.8, 496, 496.0<br>ICD10: J44.*, J43.*, J42, J41.*<br>Medications: "roflumilast", "daliresp", "tiotropium", "spiriva", "ipratropium", "atrovent", "theophylline", "slo-bid", "slo-phyllin", "theo-dur", "theo 24", "theo24", "theo-24", "uniphyl", "salmeterol", "serevent", "formoterol", "foradil", "albuterol + ipratropium", "albuterol / ipratropium", "ipratropium bromide / albuterol sulfate", "ipratropium bromide with albuterol sulfate", "combivent", "duoneb", "albuterol", "proventil", "proair", "ventolin", "fluticasone", "salmeterol", "advair", "budesonide/formoterol", "budesonide / formoterol", "budesonide - formoterol", "budesonide-formoterol", "mometasone/formoterol", "mometasone / formoterol", "dulera", "beclomethaosne", "qvar", "budesonide", "pulmicort", "fluticasone", "flovent", "mometasone", "asmanex"
CKD	Presence of ANY eGFR < 60 ml/min throughout the record

CAD: Coronary artery disease, COPD: Chronic obstructive pulmonary disease, CKD: Chronic kidney disease.

© 2021 Davogustto GE et al. *JAMA Network Open.*

eTable 2. Organ System Involvement Definitions

Organ System involvement	Definition (any from the list)
Cardiovascular	- Arrhythmia (excluding sinus tachycardia and sinus bradycardia)<br>- New LVEF < 55% or new WMA<br>- Pulmonary edema due to left sided heart failure<br>- Pericarditis or pericardial effusion (> trivial)<br>- BNP > 400 pg/ml<br>- TnI > 0.03ng/ml<br>- Definite Myocarditis[a]<br>- Probable Myocarditis[b]<br>- Possible Myocarditis[b]<br>- Hypotension or shock<br>- Vasopressor or inotropic requirement<br>- Mechanical circulatory support requirement<br>- Cardiopulmonary Resuscitation
Nervous system	- Stroke or acute intracranial hemorrhage<br>- Seizures<br>- Suspected CNS infection<br>- Coma<br>- Encephalitis, aseptic meningitis, or demielynating disorder diagnosed by a neurologist<br>- Decreased hearing or vision<br>- Iritis or uveitis
Mucocutaneous	- Bilateral conjunctival injection<br>- Oral mucosa changes<br>- Peripheral extremity changes<br>- Skin rash or ulcers<br>- “COVID toes”<br>- Swollen red cracked lips<br>- Erythema of palms or soles<br>- Edemas of hands and feet<br>- Periungual descamation<br>- Conjunctivitis<br>- Peripheral gangrene
Gastrointestinal	- Nausea and/or vomiting<br>- Diarrhea<br>- Abdominal pain<br>- Appendicitis<br>- Pancreatitis<br>- Hepatitis (AST or ALT > 40 IU/L)<br>- Gallbladder hydrops or edema<br>- Hepatomegaly or splenomegaly
Renal	- AKI[c]<br>- Hyponatremia (<135 mmol/L)<br>- Proteinuria (Any + on UA)<br>- Dialysis requirement
Respiratory	- Severe bronchospasms requiring bronchodilators<br>- Pulmonary infiltrates on CXR or CT<br>- Lower respiratory tract infection<br>- Pleural effusion<br>- Pneumothorax<br>- Pulmonary hemorrhage<br>- Chest tube or drainage required

© 2021 Davogustto GE et al. *JAMA Network Open.*

Hematologic	- Leukopenia (total WBC < $4x10^3/\mu l$)<br>- Neutrophilia (>7.7 $x10^3$ neutrophils/µl)<br>- Lymphopenia (<1 $x10^3$ lymphocytes/µl)<br>- New hemoglobin < 9 gr/dl<br>- Thrombocytopenia (< 150 $x10^3/\mu l$)<br>- Major bleeding[d]<br>- Minor bleeding[e]<br>- VTE<br>- Arterial thromboembolism<br>- Hemolysis
Musculoskeletal	- Arthritis or arthralgia<br>- Myositis or myalgia
Other	- Any organ involvement not specified above but that triggered clinical intervention, i.e. testing and/or treatment.

Modified from Feldstein LR et al.[3] LVEF: Left ventricular ejection fraction, WMA: Wall motion abnormality, TnI: Troponin I, AKI: Acute kidney injury, UA: Urinalysis, CXR: Chest X ray, CT: Computed tomography, WBC: White blood cell count, VTE: Venous thromboembolism. [a]Definite myocarditis by endomyocardial biopsy, cardiac magnetic resonance, or as defined by the European Society of Cardiology.[5] [b]Probable and Possible myocarditis as defined by the European Society of Cardiology.[5] [c]Acute kidney injury as defined by KDIGO clinical practice guidelines.[6] [d]Fatal bleeding or symptomatic bleeding in a critical area (intracranial, intraspinal, intraocular, retroperitoneal, intraarticular, pericardial, intramuscular with compartment syndrome) or causing hemoglobin decrease ≥ 2.0 gr/dl or requiring transfusion ≥ 2 packed red blood cells.[7] [e]Any bleeding leading to or during admission not meeting criteria for major bleeding.[7]

© 2021 Davogustto GE et al. *JAMA Network Open.*

eFigure 1. Overview of Identification of Patients Meeting Multisystem Inflammatory Syndrome in Adults (MIS-A) Case Definition

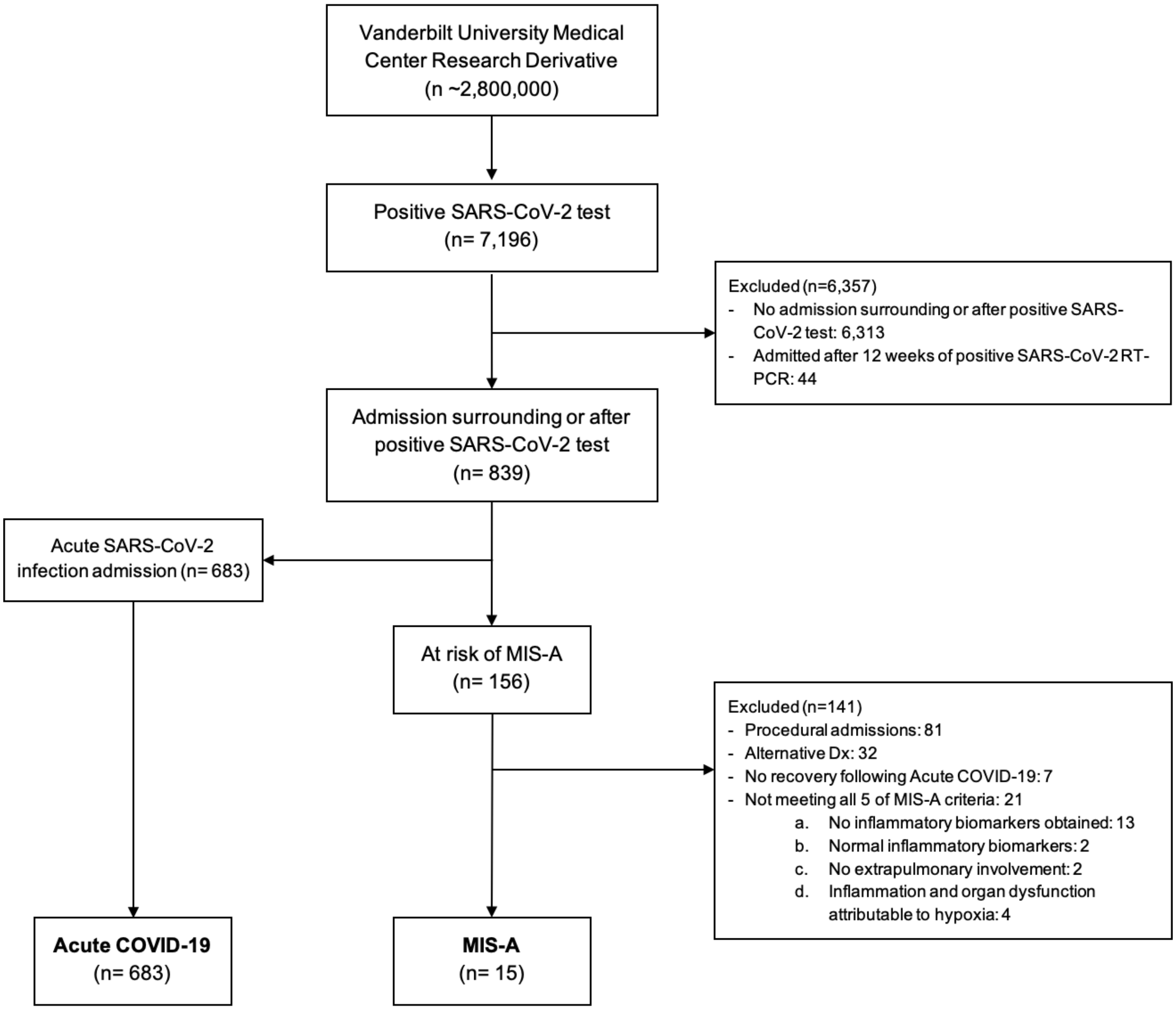

MIS-A: Multisystem inflammatory syndrome in adults, RT-PCR: Reverse transcription polymerase chain reaction, Dx: Diagnosis.

© 2021 Davogustto GE et al. *JAMA Network Open.*

eFigure 2. Identification of Participants at Risk of Multisystem Inflammatory Syndrome in Adults (MIS-A) From the Electronic Health Record

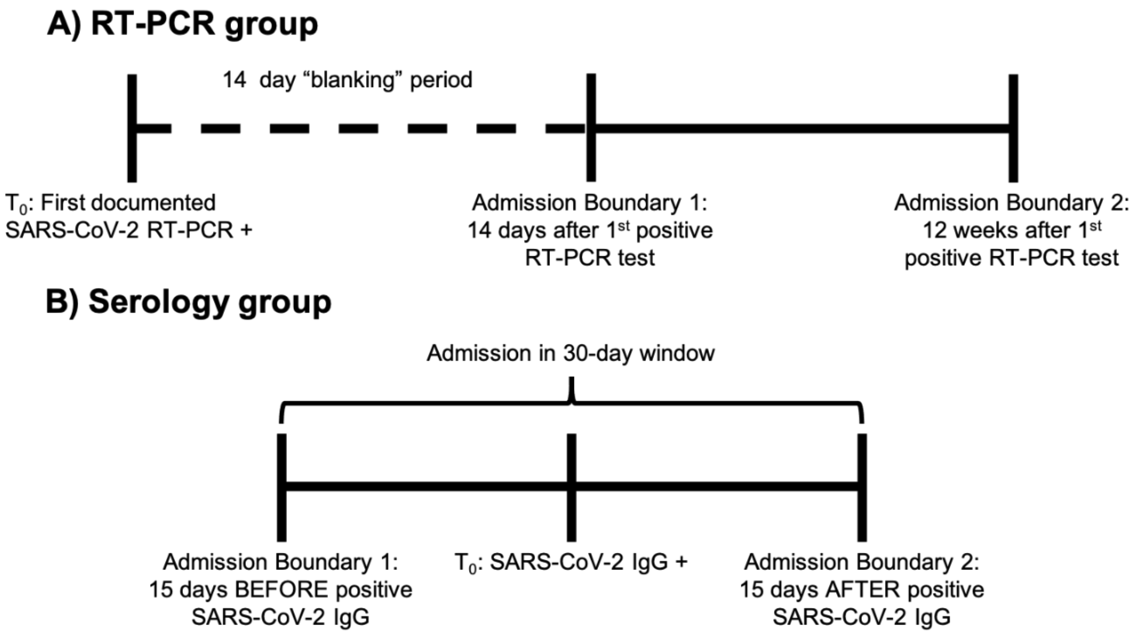

RT-PCR: Reverse transcription polymerase chain reaction, IgG: Immunoglobulin G.

© 2021 Davogustto GE et al. *JAMA Network Open.*
